# Production Strategies for Pentamer-Positive Subviral Dense Bodies as a Safe Human Cytomegalovirus Vaccine

**DOI:** 10.3390/vaccines7030104

**Published:** 2019-09-01

**Authors:** Patricia Gogesch, Inessa Penner, Steffi Krauter, Nicole Büscher, Leander Grode, Inci Aydin, Bodo Plachter

**Affiliations:** 1 Institute for Virology, University Medical Center of the Johannes Gutenberg-University Mainz, D-55131 Mainz, Germany; pgogesch@uni-mainz.de (P.G.); inpenner@uni-mainz.de (I.P.); krauter@uni-mainz.de (S.K.); bueschni@uni-mainz.de (N.B.); 2 Vakzine Projekt Management GmbH, Mellendorfer Str. 9, D-30625 Hannover, Germany; grode@vakzine-manager.de (L.G.); aydin@vakzine-manager.de (I.A.)

**Keywords:** cytomegalovirus, vaccine, dense bodies, congenital infection, safety vector, pentamer complex, gH/gL/UL128-131

## Abstract

Infections with the human cytomegalovirus (HCMV) are associated with severe clinical manifestations in children following prenatal transmission and after viral reactivation in immunosuppressed individuals. The development of an HCMV vaccine has long been requested but there is still no licensed product available. Subviral dense bodies (DB) are immunogenic in pre-clinical models and are thus a promising HCMV vaccine candidate. Recently, we established a virus based on the laboratory strain Towne that synthesizes large numbers of DB containing the pentameric protein complex gH/gL/UL128-131 (Towne-UL130repΔGFP). The work presented here focuses on providing strategies for the production of a safe vaccine based on that strain. A GMP-compliant protocol for DB production was established. Furthermore, the DB producer strain Towne-UL130rep was attenuated by deleting the UL25 open reading frame. Additional genetic modifications aim to abrogate its capacity to replicate in vivo by conditionally expressing pUL51 using the Shield-1/FKBP destabilization system. We further show that the terminase inhibitor letermovir can be used to reduce infectious virus contamination of a DB vaccine by more than two orders of magnitude. Taken together, strategies are provided here that allow for the production of a safe and immunogenic DB vaccine for clinical testing.

## 1. Introduction

The human cytomegalovirus (HCMV) is well-recognized as a clinically important pathogen. Transmission of the virus during pregnancy and the resulting congenital HCMV infection (cCMV) are frequently associated with severe sequelae [1,2,3]. The development of a vaccine against cCMV has thus been defined as a top-priority medical goal [4,5]. Additionally, HCMV reactivation is a severe complication of both solid organ and hematopoietic stem cell transplantation [6,7]. The establishment of a vaccine for the prevention of HCMV-related complications in these settings is highly desirable [8].

Several vaccine candidates are currently being tested in pre-clinical or clinical studies (reviewed in [9]). However, there is still an ongoing debate with regard to the goals and the appropriate formulations of a vaccine (reviewed in [9,10,11,12,13,14]). The tegument protein pp65 (pUL83) and the immediate-early protein 1 (IE1, pUL123) have gained broad endorsement as being major T lymphocyte antigens to be included in a vaccine. Lesser consensus has been reached regarding the viral proteins that may be necessary to induce protective humoral immune responses following vaccination. The glycoproteins gB (gpUL55) and gH (gpUL75) have been identified as prominent targets of neutralizing antibodies (nabs) [15,16,17]. However, clinical studies have demonstrated only limited protective effects afforded by a gB subunit vaccine [18,19]. This suggests that additional antigens might be needed to induce sufficient antibody levels for protection against infection. The pentameric protein complex (PC) of HCMV envelope proteins, consisting of gH, gL, and pUL128-131, has been identified as a crucial component of the HCMV virion that mediates viral entry into a broad spectrum of host cells, including epithelial cells, endothelial cells (EC), and dendritic cells [20,21,22]. The PC has also been found to be a major target of the humoral response, as a large proportion of the nabs capacity in convalescent human sera has been found to be directed against this complex. These findings support the concept of including the PC as a component of a future HCMV vaccine [23,24].

**Figure 1:**
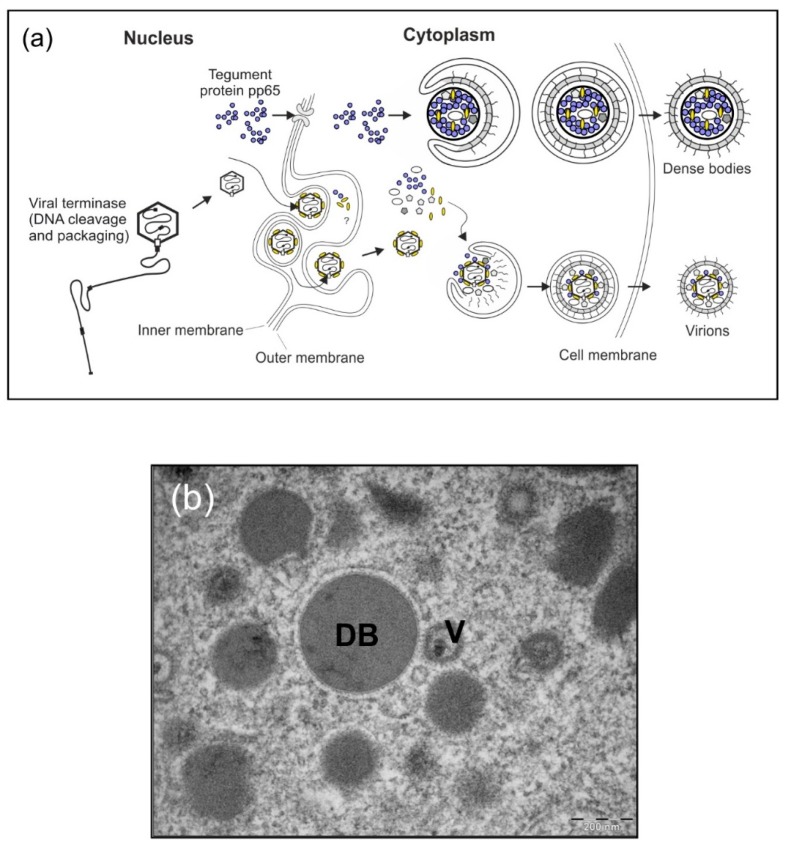
HCMV-infected cells shed progeny virions as well as DB. (**a**) Schematic model of virus and DB production in HCMV-infected cells. During the infectious cycle of HCMV, novel genomes are synthesized in the cell nucleus as concatemers. The cleavage and packaging of these large DNA molecules into capsids are mediated by the viral terminase. Tegumentation is likely initiated already prior to capsid-egress through the nuclear membranes and continues in the cytosol, where finally the capsid-tegument complexes are enveloped and secreted into the extracellular space as progeny virions (lower section). Simultaneously, the viral tegument protein pp65 and a selected set of other tegument proteins are exported from the nucleus where they assemble together with cytoplasmic tegument proteins to form subviral particles, termed DB. Similar to infectious virions, DB are enveloped and released (upper section). The envelope of DB is fusogenic and thus very likely contains viral envelope proteins in their functional conformation, comparable to infectious virions. DB are devoid of viral DNA. (**b**) Electron micrograph of HCMV-infected human foreskin fibroblasts (HFF) which contain virions (V) and cytoplasmic DB.

One vaccine candidate that has been studied in our laboratory and by others is based on subviral particles of HCMV, known as dense bodies (DB) [25,26,27,28,29,30,31,32,33,34,35] (Table 1). DB are synthesized in infected fibroblast cell cultures and are released from these cells at late stages of HCMV replication, concomitant with the release of virions [36,37] (Figure 1). DB are devoid of viral capsids and DNA and are therefore non-infectious [38]. The internal structure of DB mainly consists of pp65 and other tegument proteins [27,37,38,39]. This electron-dense core is enclosed by a phospholipid bilayer which includes the major viral envelope protein complexes. These complexes are likely inserted into the DB-membrane in a fusion-competent conformation, as they mediate swift entry into cells [40]. Consequently, antibodies induced by DB application will likely also be suitable to target envelope complexes of infectious virions in their pre-fusion conformation, thereby preventing viral entry into cells.

**Figure 2:**
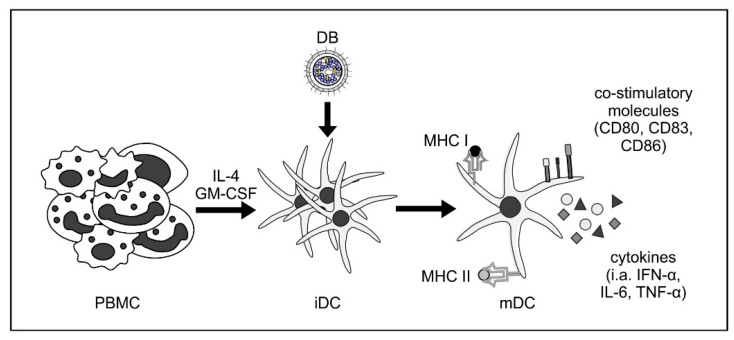
DB induce activation and maturation of monocyte-derived immature dendritic cells. Human immature dendritic cells (iDC) were generated by the incubation of peripheral blood mononuclear cells (PBMC) with interleukin-4 (IL-4) and granulocyte macrophage-colony stimulating factor (GM-CSF). Incubation of these iDC with DB induces their maturation towards a myeloid DC (mDC) phenotype, as measured by flow cytometry (CD80, CD83, and CD86). These DC were also activated by DB incubation, as shown by the increase of cytokine secretion (i.a., IFN-α, IL-6, and TNF-α) and increased expression of major histocompatibility complexes (MHC) class I and II [28].

DB induce both CD4- and CD8-T lymphocyte responses when applied to animals [29,32,34]. This is a remarkable feature considering the fact that DB do not replicate following application. In addition, these responses were achieved without the addition of an adjuvant. DB application also induces distinct nabs responses against HCMV infection [29,32,33,34]. The impressive immunological properties are likely related to the capacity of DB to induce both activation and maturation of immature dendritic cells (DC; Figure 2) [28]. The potential of DB as an HCMV vaccine has however been challenged by the fact that most studies regarding the immunogenicity of these particles have been performed with laboratory strains of the virus. DB from these strains lack the PC. As mentioned above, the inclusion of the PC in a prospective vaccine is likely important for its efficacy. DB from laboratory strains do not express the PC due to mutations in the genes encoding UL128-131. We have recently genetically modified a derivative of the laboratory strain Towne to restore expression of the PC [35]. DB purified from the culture supernatant of primary human foreskin fibroblasts (HFF) infected with this strain contain the functional PC, as these particles enter both fibroblasts and endothelial cells. Immunization experiments in mice and rabbits with these PC-positive particles have shown that the sera of the animals had higher neutralization capacities against HCMV infection in EC and fibroblasts compared to the response following immunization with PC-negative DB [35]. Consequently, the modified Towne strain, denominated Towne-UL130repΔGFP, provides an excellent basis to develop a production process for a DB-based vaccine. In this communication, we will discuss some of the aspects for the production of a safe DB vaccine for initial clinical studies.

## 2. Materials and Methods

### 2.1. Cells, Bacterial Artificial Chromosome (BAC)-Cloning, and Viruses

HFF were cultured as described previously [41]. All HCMV strains used in this analysis were derived from BAC clones. For downstream cloning within this study, the recently established parental strain Towne-UL130repΔGFP [35] (hereafter referred to as Towne-repΔGFP) was used. Human umbilical vein endothelial cells (HUVECs) conditionally immortalized with tetracycline-dependent expression of the SV40 large-T antigen and hTERT (HEC-LTT) were cultured as described previously [42,43]. For growth, HEC-LTTs were cultured in endothelial cell growth medium (EGM BulletKit, Lonza, Basel, Switzerland) supplemented with 2 mg/mL doxycycline (Applichem, Darmstadt, Germany). Cloning procedures were performed based on the bacterial galactokinase (GalK) positive/negative selection as described by Warming et al. [44]. Strains HCMV-UL51-FKBP and HB5 were kindly provided by Eva Borst and Martin Messerle [45].

For the generation of Towne-UL130rep-GalK-KO (hereafter referred to as Towne-rep-GalK-KO), the GalK-gene, which initially was inserted for the depletion of GFP in the BAC cassette, was seamlessly deleted by recombination with a synthetic DNA fragment consisting of the HCMV-derived homologies flanking GalK. This resulted in the depletion of GalK without insertion of additional sequences.

For the generation of Towne-UL130rep-dUL25 (hereafter referred to as Towne-rep-ΔUL25), the UL25-gene of the parental strain Towne-rep-GalK-KO was replaced by a GalK-cassette which was amplified from the plasmid pGalK [44]. We used primers comprising 50 base pairs of sequences homologous to the genomic region flanking the UL25 gene (Towne_UL25-GalK_fwd: ACCGGCGCCGCCAAGAAACCGAGCGAAAAGAAACGATCGTCGTCGCGTCGCCTGTTGACAATTAATCATCGGCA, Towne_UL25-GalK_rev: CCTGTGACTTTTTATCATAAACCGTTCCGC CCTGCTGCTTCGTTCCACCATCAGCACTGTCCTGCTCCTT).

Virus reconstitution from BAC-clones was achieved by transfecting column-purified BAC-DNA (Plasmid Purification Kit; Machery&Nagel, Düren, Germany) into HFF with Superfect transfection reagent (Qiagen, Hilden, Germany) as described previously [25]. Viral stocks were generated by passaging transfected HFF until all cells showed a typical cytopathic effect. The supernatants were then collected and used as seed stocks. Supernatants were frozen at −80 °C until further use. Viral stocks of HCMV-UL51-FKBP were generated in the presence of Shield-1 (1 µM, supplemented every 48 h, Aobious, Köln, Germany).

### 2.2. Production and Purification of Virions and DB

Virions and DB of HCMV were prepared as previously described [25]. HFF were infected with culture supernatants containing the virus of interest. For the letermovir experiments, 50 nM or 300 nM of the substance were added to the cell culture during infection and 3 days after initial infection. Culture supernatants from infected HFF were collected 1 week after infection, and, after removal of cellular debris, pelleted via ultracentrifugation. After resuspension, the different components of the resulting pellet were fractionated via glycerol-tartrate density gradient ultracentrifugation [37]. Subsequently, virions and DB were isolated, concentrated, and stored at −80 °C until further use. For the production of HCMV-UL51-FKBP-derived DB, HFF were infected with HCMV-UL51-FKBP in the initial presence of Shield-1 (1 µM) to allow viral spread through the complete cell culture. After 3.5 days, the Shield-1-containing medium was replaced with Shield-1-free medium to inhibit synthesis of infectious virus while retaining DB production. Supernatants of the cells were harvested 1 week after initial infection and processed as described above.

### 2.3. SDS-PAGE, Silver/Instant Blue Staining, and Immunoblotting

The protein composition of purified virions and DB was analyzed by SDS-PAGE, followed by either silver staining, instant blue staining, or by immunoblotting, respectively. For silver staining, 2 µg of virions or DB per lane were loaded on a 10% tris-glycine-polyacrylamide gel. For instant blue staining, 20 µg of DB were loaded on a 4–12% bis-tris-polyacrylamide gel (Thermo Fisher Scientific, Darmstadt, Germany). For immunoblotting, 30 µg of virions or DB per lane were loaded on a 10% bis-tris-polyacrylamide gel.

For silver staining, SDS-Gels were fixed and processed with the Roti^®^-Black P silver staining kit for proteins (Carl Roth, Karlsruhe, Germany). For instant blue staining, the SDS-Gel was incubated in 20 mL of the staining solution according to the manufacturer’s protocol (Expedeon via BIOZOL Diagnostica, Eching, Germany). For immunoblotting, proteins were transferred to a PVDF membrane (Immobilon-FL, Millipore, Billerica, MA, USA). Expression of the pentamer-complex proteins (UL128-131, gH, gL) on viral particles and DB was analyzed using a polyclonal PC-specific antibody raised in sheep (The Native Antigen Company, Kidlington, UK) using an anti-sheep HRP-coupled secondary antibody (The Native Antigen Company, Kidlington, UK).

### 2.4. Immunofluorescence

For indirect immunofluorescence analysis, HFF or EC (HEC-LTT) (2 × 10^5^ per well) were grown on coverslips in 6-well plates. The next day, HFF were incubated with 2 µg and EC with 10 µg of DB derived from strains Towne-BAC or Towne-repΔGFP, respectively. After 24 h, cells were handled as previously described [26]. For detection of viral pp65, the specific mouse monoclonal antibody 65-33 (kindly provided by William Britt, University of Birmingham, Birmingham, AL, USA) was used. Nuclei were stained with 4′,6-Diamidin-2-phenylindol (DAPI) and analyses were performed with a Leica DM IRB microscope.

### 2.5. Analysis of Infectious Virus by IE1-Staining

The determination of residual infectivity within DB preparations was performed by staining with monoclonal antibody 63-27, directed against IE1 [46,47], provided by William Britt. For this, 5 × 10^3^ HFF per well were infected with tenfold serial dilutions of DB preparations (1 µg/mL). Forty-eight hours after infection, cells were washed in PBS and fixed with 96% ethanol for 20 min at room temperature. After a further washing step, cells were incubated for 1 h at 37 °C with 50 µL hybridoma supernatant of monoclonal antibody p63-27. Binding of the IE1-specific antibody was detected with a horse-radish peroxidase (HRP)-coupled polyclonal rabbit anti-mouse secondary antibody (Agilent, Waldbronn, Germany). Antibody binding was visualized by incubation with a 3-amino-9-ethylcarbazole (AEC) solution. After another washing step, the numbers of the IE1-positive nuclei were counted in the microscope. The mean of 8 technical replicates was taken as the relative measure of infectivity.

### 2.6. IFN-β Treatment and Analysis of Genome Replication Kinetics

Subconfluent HFF were treated with IFN-β (100 U/mL diluted in 0.1% bovine serum albumin–double-distilled H_2_O; specific activity, according to the manufacturer’s information, 5 × 10^8^ U/mg; catalog number 300-02BC; PeproTech, Hamburg, Germany) or left untreated as a control. After 12 h of incubation, the cells were infected with 50 genome copies/cell of Towne-repΔGFP or Towne-repΔUL25. To measure intracellular and extracellular viral genomes, DNA from 1 × 10^5^ infected cells or 200 µL of cell culture supernatants was isolated, respectively, using the High Pure viral nucleic acid kit (Roche, Mannheim, Gemany). The amount of genome copies was determined by HCMV-specific TaqMan PCR analysis using an ABI 7500 Fast real-time PCR detection system measuring triplicate technical replicates with the probe 5′-6-carboxyfluorescein-CCACTTTGCCGATGTAACGTTTCTTGCAT-tetramethyl-rhodamine (fwd primer: TCATCTACGG GGACACGGAC; rev primer: TCATCTACGGGGACACGG AC).

### 2.7. Statistical Analyses

Statistical analyses were performed using GraphPad Prism version 4.2.4. (GraphPad Software Inc., San Diego, CA, USA).

## 3. Background, Results, and Strategy for Development

### 3.1. Background

DB contain major HCMV antigens and have been shown to be highly immunogenic without the addition of adjuvant [27,28,29,32,33,34,39]. Remarkably, DB contain major viral envelope proteins in their pre-fusion conformation, providing important antigens for the induction of a nabs response. One obstacle in the course of generating DB for clinical studies relates to the fact that the synthesis of these particles requires viral infection of culture cells. Although separating DB from virions is achieved by glycerol-tartrate gradient centrifugation of culture supernatants [37], DB fractions are still potentially contaminated by infectious virus. Thus, a large-scale GMP-compliant production process, which includes efficient inactivation of residual infectivity in DB material, was developed here. In addition, this work focused on reducing the viral load in culture supernatants used for DB purification and on the establishment of an attenuated HCMV seed strain that would be replication incompetent in vivo.

### 3.2. Results and Strategy for Development

#### 3.2.1. HCMV DB Material Can be Produced in a Large-Scale GMP-Compliant Manner

**Figure 3:**
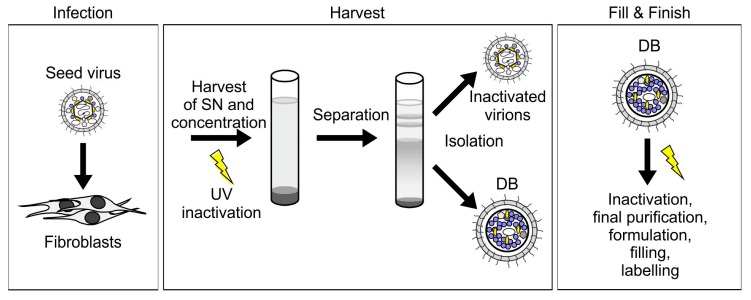
The purification procedure for HCMV-derived DB is scalable to a GMP-compliant vaccine production protocol. For the production of DB as an HCMV-vaccine, fibroblasts are infected with the seed virus (infection). Cell culture supernatants (SN) are harvested and pelleted via ultracentrifugation. UV-irradiation is performed for virion inactivation. DB and virions are then separated by density gradient ultracentrifugation and isolated (harvest). Fill & Finish comprises further inactivation via gamma-irradiation, purification, formulation, filling, and labelling.

Infection of human culture fibroblasts with HCMV results in the production and release of progeny virions and DB (Figure 1). Hence, HCMV-permissive cells (e.g., human fibroblasts as in our protocol) have to be infected with the respective seed virus prior to recovery of viral stocks or of DB material for vaccine preparation. A feasibility study analyzing the generation of HCMV DB in human fibroblasts using our model seed virus has been was successfully performed. Consequently, a large-scale, GMP-compliant production system has been developed based on the DB isolation procedure established in our lab (Figure 3). The de novo synthesized virus and DB particles are secreted into the cell culture supernatant. This material was isolated (harvest) and further processed for vaccine preparation. The next steps included UV-irradiation of the harvested, cell-free supernatant to inactivate infectious viral particles, which are co-secreted by the infected cells. The UV inactivation step during the production of the DB-vaccine was performed in an automatic tube reactor via dynamic UVC irradiation adjusted for the absorbance of each clarified supernatant (UVivatech Bayer Technology Services Leverkusen, Germany) with 1000 J/m^2^ for efficient inactivation with a tolerable aggregation rate. Five DB-sub-batches generated using the established procedures showed <500 infectious units per mL before gamma irradiation, which met the acceptance criteria for release for this parameter. Concentration of this material was followed by density-gradient ultracentrifugation to separate virions and DB. DB fractions were subsequently processed for downstream vaccine production. For this, a second inactivation step by gamma-irradiation was performed during final purification, formulation, filling, and labelling (Fill & Finish). Preliminary studies with regards to long term storage properties showed that DB produced in this system were biochemically and biophysically stable at −20 °C for up to three months. Additionally, immunization of mice with this material verified the immunostimulatory capacity of the DB. In these studies, DB were suitable to induce both cytotoxic T lymphocyte responses as well as HCMV-specific antibody responses. With the establishment of MRC5 cultures in the FibraStage cultivation systems, a large-scale start volume could be processed for final bulk preparation, filling 96 vials with 1.15 mL of the HCMV-DB preparation (0.22 mg/mL). Conduction of the established release assays resulted in successful batch-testing and release of DB material suitable for toxicological tests. Preliminary pyrogenicity analyses in rabbits were performed showing a minimal rise in temperature far below the respective acceptance criteria. Taken together, this provides the basis for further up-scaling to a large-scale GMP-compliant production process suitable for the generation of material for clinical testing.

#### 3.2.2. Further Development of Pentamer-Positive DB

The viral antigens that are packaged into DB have to be encoded by the seed virus used for production. This includes all components of the PC. It is well accepted that the PC is a critical antigenic component that has to be considered for HCMV vaccine-design [9,48,49,50]. HCMV laboratory strains are high-level DB producers and yet they are devoid of the PC. On the contrary, recent clinical isolates express the PC, but they are low-level DB producers. The amount of DB, released from HFF after infection with TB40 or comparable isolates is insufficient for upscaling (Büscher et al., unpublished). Approaching this discrepancy, we have recently established a pentamer positive (PC^+^) variant of the laboratory strain Towne (Towne-repΔGFP, [35]). The original Towne strain carries a mutation in the UL130 gene, abrogating the synthesis of a functional PC. We repaired the mutation by replacing the Towne UL130-gene with the UL130-gene of strain TB40/e, using BAC mutagenesis [35]. The PC components gH, gL, and pUL131 were detectable by Western blot analyses of Towne-repΔGFP, using a commercially available polyclonal antiserum for detection.

**Figure 4:**
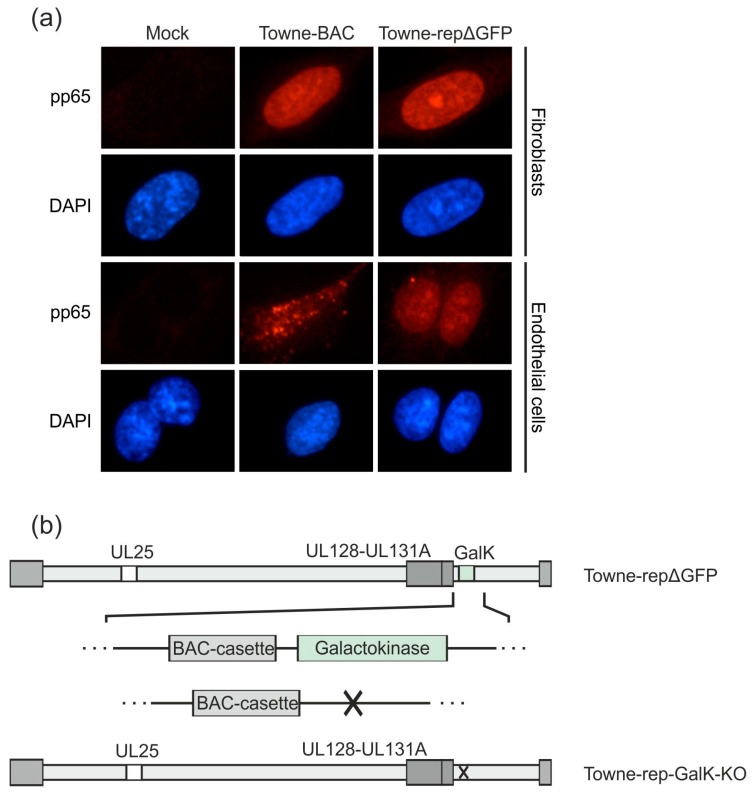
Downstream development of an HCMV strain Towne derivative with a functional pentamer complex. A UL128-131-gH-gL competent DB producer strain, based on the laboratory strain Towne, has been established previously (Towne-repΔGFP; [35]). (**a**) HFF or endothelial cells were incubated with 2 µg (HFF) or 10 µg (endothelial cells) DB of Towne-repΔGFP or its parental pentamer-defective strain Towne-BAC and analyzed by immunofluorescence microscopy 1 day post application (magnification of 400). Nuclei were stained with DAPI (blue). The localization of DB-derived pp65 was detected using a pp65-specific monoclonal antibody and an anti-mouse Alexa Fluor^®^ 546-conjugate secondary antibody (red). (**b**) The galactokinase (GalK) expression cassette, which was inserted for the deletion of a GFP-sequence in the BAC-cassette in Towne-repΔGFP, was deleted by homologous recombination. The resulting strain was termed Towne-rep-GalK-KO and was used as the basis for the further development of a seed virus strain.

To demonstrate that a functional PC was expressed on the surface of DB from strain Towne-repΔGFP, EC were incubated with these particles. EC can only be penetrated by DB when the PC is expressed on their surface in its functional conformation. EC and, for control, HFF were incubated with purified Towne-repΔGFP-derived DB and Towne-BAC-derived DB (Figure 4a). Cells were incubated for 24 h with DB and were then collected for indirect immunofluorescence analysis. The uptake of the major tegument protein pp65 was analyzed by a specific monoclonal antibody and was taken as surrogate for entry. Both Towne-repΔGFP-derived DB and Towne-BAC-derived DB entered HFF with comparable efficiency, as evidenced by the nuclear staining for pp65. This was expected, as entry into HFF is PC-independent. By contrast, pp65 was exclusively detectable in the nuclei of those EC cultures that had been exposed to Towne-repΔGFP-derived DB. Roughly 30–40% of EC showed nuclear pp65 staining. By contrast, no nuclear pp65 staining was detectable in EC cultures that were exposed to Towne-BAC-derived DB. The fuzzy staining of pp65 on the margin of the cells suggests that the Towne-BAC-derived DBs adsorbed to the surface of EC but were not suitable to enter the cells. Further analyses are required to investigate this in further detail. These experiments showed that the PC on DB from strain Towne-repΔGFP was expressed in a functional pre-fusion conformation. Consequently, Towne-repΔGFP was chosen for further seed virus development.

As a next step, we deleted the GalK-gene, which was inserted to replace the GFP-gene, from the respective BAC of that strain. The resulting BAC was denominated Towne-rep-GalK-KO (Figure 4b). This manipulation was necessary to enable further modification of that strain (see below).

#### 3.2.3. Establishment of a UL25-Deleted Virus Strain for DB Production

The production process outlined in Figure 3 enables reliable inactivation of residual infectious virus from DB preparations. To add additional safety levels, we generated a virus strain which was deleted in the genomic region of HCMV encoding pUL25, based on the BAC Towne-rep-GalK-KO. The pUL25 protein is one of the most abundant constituents of the viral tegument [39,51]. It is, however, non-essential for HCMV replication in HFF cultures [25,52]. In a previous study with the unrepaired parental Towne strain, the deletion of the UL25 open reading frame surprisingly did not reduce DB release from infected HFF. The UL25-negative virus was, however, more sensitive to IFN-β compared to the parental strain [25]. This suggests that a UL25-deleted virus is more susceptible to innate immune defense mechanisms of the host.

**Figure 5:**
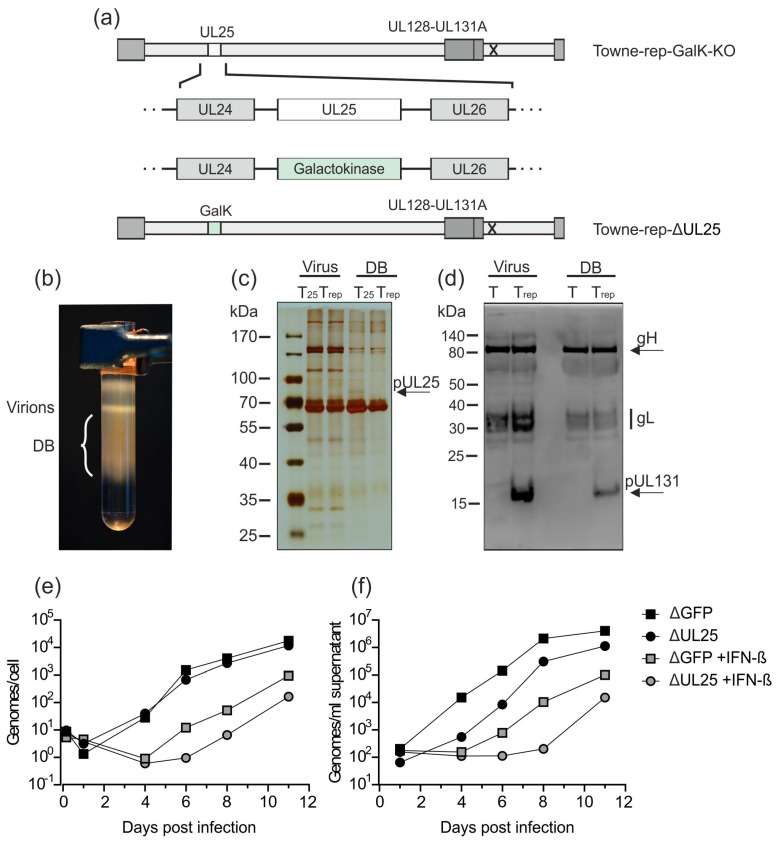
Deletion of UL25 in Towne-rep-GalK-KO does not impair the production of DB. (**a**) UL25 has been deleted in Towne-rep-GalK-KO by insertion of a GalK expression cassette via homologous recombination. The resulting strain was termed Towne-rep-ΔUL25. (**b**) HFF were infected with Towne-rep-ΔUL25. Seven days after infection, supernatants were processed for virion and DB isolation via the established procedure. A glycerol-tartrate gradient following ultracentrifugation of the cell culture supernatant is shown. Virion and DB fractions are indicated. (**c**) The virions and DB (2 µg) shown in (b) were analyzed by SDS-PAGE and silver staining to visualize the protein composition of the preparation. The virions and DB of Towne-rep-ΔUL25 (Trep) were compared to virions and DB of the UL25-expressing parental strain (T25) (expected size of pUL25 marked by an arrow). (**d**) Immunoblot analyses of 30 µg virions and DB from Towne-repΔUL25 (Trep) as compared to virions and DB of the pentamer defective strain Towne-ΔUL25 (T). gH, gL, and UL131 (marked by an arrow) were detected according to their respective molecular masses using a polyclonal PC-specific antibody produced in sheep and an anti-sheep HRP-coupled secondary antibody. (**e**,**f**) Impact of IFN-β treatment on genome replication (e) and virus release (f). HFF were incubated with IFN-β or left untreated and subsequently infected with 50 genome copies per cell of Towne-repΔGFP or Towne-repΔUL25. DNA from 10^5^ infected cells (e) or 200 µL cell culture supernatant (f) was isolated and the amount of genome copies was determined by HCMV-specific TaqMan analysis.

As such an attenuation may be an attractive feature of a seed virus, used for DB production, Towne-rep-GalK-KO was modified according to the previously published strategy for deletion of UL25 (Figure 5a) [25]. The bacterial GalK gene was inserted into the UL25 genomic region, thereby deleting this gene. The resulting strain, obtained after reconstitution in HFF, was termed Towne-rep-ΔUL25. To test if this strain was capable of producing DB in sufficient amounts, HFF were infected. Supernatants were collected 1 week after infection and DB and virions were isolated by glycerol-tartrate gradient centrifugation. A distinct DB fraction was visible in the gradient from the Towne-rep-ΔUL25 purification (Figure 5b). Polyacrylamide-gel electrophoresis and subsequent silver staining revealed protein patterns in both virions and DB from Towne-rep-ΔUL25 that were indistinguishable from those of the parental strain (Figure 5c). The only difference seen was the lack of pUL25 in the particles of the mutant. Note that pUL25 is enriched in DB. Consequently, the lack of pUL25 is more easily detectable in the DB preparations of Towne-rep-ΔUL25. To confirm that components of the PC were retained in Towne-rep-ΔUL25, immunoblot analyses were performed (Figure 5d). The results showed that gH, gL, and pUL131 of the PC were present in Towne-rep-ΔUL25 DB, indicating that the PC was contained in the particles of that strain. The unrepaired control strain Towne-BAC did not show an equivalent signal for pUL131. Note that neither pUL128 nor pUL130 could be detected in Towne-rep-ΔUL25 virions and DB using this antiserum. Since these proteins were also undetectable in virions of the TB40/e strain, antibodies against pUL128 and pUL130 appeared to be contained in that antiserum in low levels. pUL131 was undetectable in the PC^−^ parental strain Towne-BAC as expected. Both gH and gL were found as they are components of the trimeric gH/gL/gO complex expressed by both PC^−^ and PC^+^ viruses.

To confirm that the deletion of UL25 in Towne-rep-ΔUL25 resulted in increased susceptibility towards type I IFN, IFN-β-treated HFF or untreated control-cells were infected with 50 genome copies/cell of Towne-rep-ΔGFP or Towne-rep-ΔUL25. DNA preparations from cell lysates and supernatants were collected at indicated time points and subjected to quantitative TaqMan PCR analysis (Figure 5e,f). Both strains showed reduced genome replication and release of viral DNA when the cells were treated with IFN-β. The experiments were confirmed using an infectious dose of 100 genome copies/cell. These results confirmed that HCMV progeny production was impaired by IFN-β. Remarkably, Towne-rep-ΔUL25 showed more pronounced IFN-β-mediated inhibition of replication compared to Towne-rep-ΔGFP, indicating attenuation and increased sensitivity of that strain to innate immune responses through the deletion of pUL25.

#### 3.2.4. Application of the Terminase Inhibitor Letermovir in the DB Production Process

HCMV-derived DB are complex structures which can only be generated in cell culture in an infectious system. Thus, with regards to vaccine production, it is a major task to ensure complete removal of residual infectivity. The two irradiation steps, which are already included in the production process (Figure 3), have been shown to abrogate residual infectivity. As outlined above, however, we have focused our attention on implementing additional levels of safety in the production process.

**Figure 6:**
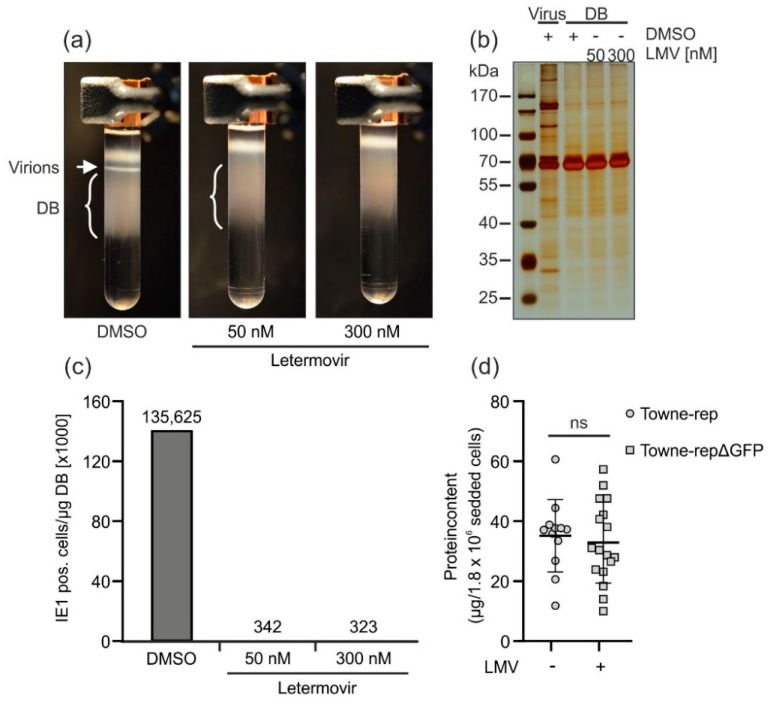
Application of letermovir during DB production reduces the secretion of progeny virions. HFF were infected with laboratory strain AD169 (RV-HB5) in the presence of the indicated concentrations of letermovir. The solvent DMSO was used as a negative control. Seven days post infection, supernatants were processed for virion and DB isolation via glycerol-tartrate gradient ultracentrifugation. (**a**) Glycerol-tartrate gradients following ultracentrifugation of the cell culture supernatant are shown. Virion fractions and DB fractions are indicated. Note the lack of the virion band in letermovir-treated samples. (**b**) The virion and DB fractions as shown in (a) were analyzed by SDS-PAGE and silver staining to visualize their protein composition. DB isolated in the absence or presence of levermovir (LMV) were compared to virions isolated in the absence of letermovir. (**c**) Residual infectivity was determined by incubation of HFF with a tenfold serial dilution of a 1 µg/µL DB preparation purified in the absence or presence of letermovir as shown in (a). Forty-eight hours post incubation, cells were stained immunocytochemically for IE1 expression. IE1-positive cells were counted using a Leica DM IRB microscope. (**d**) HFF were infected with Towne-rep in the absence of letermovir or with Towne-rep-ΔGFP in the presence of letermovir. Seven days after infection, supernatants were processed for virion and DB isolation via glycerol-tartrate gradient ultracentrifugation. The absolute protein contents, as a measure for DB yield, from the preparations of letermovir treated versus untreated cultures were determined and normalized by the initial number of seeded producer cells. Several biological replicates are shown in each case. Error bars indicate the standard deviation. Legend: ns = not significant (non-paired, two-tailed Mann Whitney U-Test).

Letermovir (Prevymis^®^) is a viral terminase inhibitor that has recently been licensed for the prophylaxis of HCMV infection in hematopoietic stem cell transplant recipients [53]. The drug efficiently blocks the viral terminase which mediates the packaging of the large HCMV genome into capsids. We hypothesized that the application of letermovir during the culturing of infected HFF would significantly reduce virus release without impairing DB production, thereby providing a strategy for depletion of infectious virus from DB. To verify this, HFF were infected with the laboratory strain AD169 (RV-HB5, [54]) and grown in the presence or absence of 50 nM or 300 nM letermovir. DMSO was used as a negative control. After 1 week, virion and DB purification was performed by glycerol-tartrate gradient centrifugation. Indeed, virion fractions were undetectable in the gradients of the materials derived from those infected cells that were grown in the presence of 50 nM or 300 nM letermovir. Virions were clearly seen in the negative control (DMSO; Figure 6a). To show that the protein patterns of DB were conserved after letermovir treatment and to confirm that DMSO as a solvent of letermovir had no effect on the expression pattern of viral proteins, SDS-PAGE and silver staining were performed. As shown in Figure 6b, the DB protein patterns were indistinguishable between the samples obtained in the presence or absence of letermovir. The patterns were, in addition, comparable to those seen after routine DB preparation.

To measure the reduction of contamination by infectious virus, the different DB preparations were applied to indicator HFF cultures in serial dilutions. After two days, these cells were fixed and stained with an IE1-antibody to quantify residual infectivity (Figure 6c). In fact, both concentrations of letermovir were able to reduce infectivity more than 350-fold in comparison to the DMSO control. Note the remarkably low concentration that was required to almost completely suppress virus production. To address the question about the loss of DB productivity, the yields of DB from letermovir-treated cultures were compared with those of untreated cultures (Figure 6d). No statistically significant differences were seen, indicating that letermovir may be added to the production of DB without loss in yield. Taken together, these results showed that the viral terminase inhibitor letermovir is an attractive compound for the depletion of infectivity during DB vaccine production.

#### 3.2.5. Establishment of a Shield-1-Dependent DB Production Process

An option for the production of a safe DB vaccine is the modification of essential seed virus-genes in a way that enables their expression by conditional activation. Here, we tested whether conditional expression of the viral pUL51 protein using the Shield-1/FKBP system would allow DB production while abrogating virus release [45,55]. The Shield-1/FKBP system has been previously used by others to establish an HCMV vaccine candidate based on a replication deficient viral strain [56].

**Figure 7:**
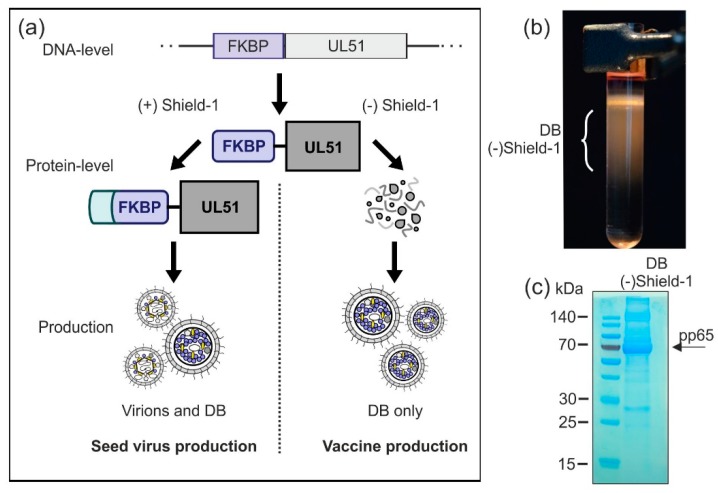
Production of virions and DB in a Shield-1-dependent system. (**a**) Schematic display of the planned Shield-1-dependent production system. In order to establish an optimized safety vector which is applicable for conditional virus and/or DB production, the Shield-1/FKBP-system is introduced to the seed virus. Endogenous pUL51, which is an essential protein for HCMV genome packaging and cleavage, is exchanged by a pUL51-variant which is fused to the destabilizing domain FKBP. Fibroblasts infected with this seed virus can only produce infectious virions when the stabilizing ligand of FKBP, Shield-1, is supplemented in the cell culture (seed-stock production). The absence of Shield-1 impairs the production of virus particles, while the secretion of DB is unaffected (vaccine production). (**b**) HFF were infected with the model-strain HCMV-UL51-FKBP [45] in the presence of Shield-1 (1 µM). Three and a half days after infection, Shield-1-containing medium was replaced with Shield-1-free medium. Seven days after infection, supernatants of the cell cultures were harvested and DB and virions were isolated by glycerol-tartrate gradient ultracentrifugation. A glycerol-tartrate gradient following ultracentrifugation of the cell culture supernatant is shown. DB fractions are indicated. (**c**) The DB as depicted in (b) were isolated and analyzed via SDS-PAGE and instant blue staining to visualize their protein composition. As indicated by the arrow, the phosphoprotein 65 (pp65) represents the main constituent of the preparation.

The envisaged strategy is outlined in Figure 7a. The essential viral protein pUL51, important for viral DNA packaging, is tagged with the F36V mutant of the 107-residue protein FKBP12 as destabilizing domain (FKBP) [45,55]. In the absence of the cell-permeable small-molecule ligand Shield-1, UL51-FKBP is unstable and thus degraded [45]. Without the expression of UL51, viral DNA cannot be cleaved and packaged. As a result, no infectious particles can be secreted in Shield-1-free cell cultures which are infected with a UL51-FKBP-expressing virus, while the production of DB should remain unaffected (vaccine production). Binding of Shield-1 to FKBP stabilizes the fusion protein and protects it from degradation, thereby restoring the function of UL51-FKBP [45,55]. Hence, in the presence of Shield-1 during cell culture, seed stocks of the safety vector may be generated (seed virus production).

In a preliminary proof-of-concept experiment, we wished to analyze whether the DB-production and release was sustained in the absence of pUL51 stabilization by Shield-1. For this we infected HFF with the test strain HCMV-UL51-FKBP [45], initially in the presence of Shield-1 to allow for viral spread in the cell culture. After the removal of Shield-1, cells were further cultured for another 4 days. Subsequently, supernatants were collected and fractionated by glycerol-tartrate gradient centrifugation. The gradient showed that DB production was sustained in the absence of Shield-1, while the virion fraction was absent (Figure 7b). The material was then subjected to SDS-PAGE and instant blue staining (Figure 7c), showing a protein pattern as expected for DB. The abundant constituent pp65 (marked with an arrow), as well as proteins corresponding to other major DB components like pp150, pp71, and pp28 were detectable. Together, these results demonstrate that HCMV-UL51-FKBP-derived DB can be produced under these conditions in the absence of Shield-1. Thus, a Shield-1/FKBP-dependent system might be applicable for the production of a DB-based HCMV vaccine. Further analyses are necessary to check the DB-yield and the level of infectivity-reduction in order to decide if this approach, in combination with the other strategies, is suitable for upscaling DB-production.

## 4. Discussion

The medical need to develop an HCMV vaccine was identified many years ago [4,5]. Several efforts have been made to establish a vaccine for both the prevention of cCMV and to attenuate the consequences of HCMV reactivation in immunosuppressed individuals since then (reviewed in [9,14,57]). Testing of some of these candidate vaccines in clinical studies has met with limited success. This may be related to findings from recent studies from communities with high HCMV seroprevalence which have indicated that approaches mimicking natural immunity may not suffice to afford complete protection against cCMV [57,58,59].

DB are a rewarding candidate to induce an immune response that is different from natural immunity and DB may thus meet the requirements for an effective vaccine. These particles are non-infectious. Consequently, they do not induce the manifold immune evasion mechanisms that are activated following natural HCMV infection or following the application of a live HCMV vaccine [60,61,62,63]. They contain large amounts of the viral antigens that are considered to be important for the induction of both humoral and cellular immunity [27,39,64,65,66]. In particular, they contain viral envelope proteins in their fusion-competent conformation which may be favorable for the induction of virus-specific protective humoral responses [67]. The exceptional antigenic potential of DB has been shown by both our laboratory and others [29,32,33,34,35] and may depend on their impact on dendritic cells, which are activated by DB exposure [29] (Figure 2).

The PC, consisting of gH/gL/UL128-131, is required for the infection of key target cells of HCMV, such as epithelial, endothelial, or dendritic cells [20,21,22]. This protein complex has received considerable attention as a target of neutralizing antibodies during natural infection, as these antibodies may bear the potential to limit infection [48,49,50,68]. We recently repaired the UL130 open reading frame in the laboratory strain Towne, enabling the reconstitution of the PC in that virus [35]. DB of Towne-rep have regained the ability for PC-dependent cell entry (Figure 4). Side-by-side immunization experiments have shown the superior potential of Towne-rep DB for the induction of neutralizing antibody responses [35]. Since the Towne strain, as opposed to clinical isolates, is a high-level DB producer, its repaired derivative, deleted for the expression of GFP (Towne-repΔGFP, Figure 4), is an attractive basis for a downstream production process of a DB-based vaccine. Remarkably, also in contrast to clinical isolates, the expression of the PC has proven to be stable during multiple passages in fibroblasts, thus enabling the establishment of a seed virus stock for vaccine production.

DB are produced on fibroblast cultures infected with a suitable seed virus strain. A production process for DB on MRC-5 fibroblasts has recently been established which includes UV irradiation to remove contaminating virus from the DB fractions prior to gradient ultracentrifugation (Figure 3). This strategy safely eliminated infectious virus from the final DB product. As the removal of pathogenic virus from a DB vaccine is, however, a fundamental requirement, we designed several additional safety strategies for DB production to provide an unimpeachably safe product for application to humans.

The attenuation of the seed virus by deletion of UL25 is one of these strategies. Removal of UL25 did not affect the efficiency of DB synthesis [25] (Figure 5b). However, the respective virus was remarkably sensitive to IFN-β [25] (Figure 5e,f). Thus, the capacity of the UL25-negative viruses to replicate in vivo will be severely attenuated. A similar strategy that will be used in parallel is the conditional expression of an essential HCMV protein in the production process. Fusion of the destabilizing FKBP-domain to the UL51 open reading frame will enable the replication of the seed virus solely in the presence of Shield-1. As DB synthesis does not require pUL51, the particles can be synthesized after initial infection in the absence of Shield-1 (see Figure 7). By contrast, viral DNA packaging and the synthesis of infectious virus will be blocked under these conditions. Thus, the expression of pUL51 under Shield-1-control will generate a safety vector for DB production that will be completely replication-incompetent in the absence of Shield-1. More work is required to evaluate if the yield of DB in the absence of Shield-1 will be sufficient for upscaling.

One additional strategy to enhance the safety of the final DB product is the addition of substances that inhibit viral replication in cell culture without hampering DB production. Inhibitors of the viral terminase complex have been identified as effective antiviral substances without grossly impairing DB synthesis [69]. Letermovir is a terminase inhibitor that has recently been licensed for prophylaxis of HCMV reactivation in hematopoietic stem cell recipients [53]. In this work we have shown that the application of letermovir reduced the viral contamination of purified DB by more than two orders of magnitude (Figure 6c). Very low concentrations of the drug (50 nM) were required to suppress virus release. Applying such low concentrations in a production process will prevent contamination of a vaccine by the drug in significant amounts. Consequently, the application of letermovir for the production of a DB vaccine is an effective strategy to reduce virus contamination.

## 5. Conclusions

Some time ago, DB were identified as a promising vaccine candidate. The addition of the PC to DB has enhanced their immunostimulatory potential. However, clinical testing is still pending. The establishment of a production process according to GMP standards has paved the way to initiate such studies. The integration of additional safety features to that production process, as outlined here, will enable the provision of a safe and immunogenic vaccine for initial clinical studies. 

## Figures and Tables

**Table 1:** Overview of publications related to dense bodies (DB).

Reference	Topic
Pepperl et al. 2000 [32]	DB induce both cellular and humoral immune responses
Pepperl-Klindworth et al. 2003 [31]	DB-mediated protein delivery
Mersseman et al. 2008 [30]	DB-mediated delivery of heterologous peptides into MHC-class I presentation
Becke et al. 2010 [29]	Induction of CD8 T cell responses against an IE1-peptide, delivered by recombinant DB
Sauer et al. 2013 [28]	Maturation and activation of monocyte-derived iDCs upon stimulation with DB (Figure 2)
Cayatte et al. 2013 [34]	Induction of broad humoral and cellular immune responses by HCMV strain Towne DB
Krömmelbein et al. 2015 [26]	Limited production of DB in CAP cells
Büscher et al. 2015 [27]	Conserved protein composition of DB of various HCMV strains
Schneider-Ohrum et al. 2016 [33]	Development of a scalable bioprocess for DB production
Lehmann et al. 2019 [35]	Pentamer-positive DB are superior to pentamer-negative DB for the induction of neutralizing antibodies
